# Development and Validation of a Nausea Severity Scale for Assessment of Nausea in Children with Abdominal Pain-Related Functional Gastrointestinal Disorders

**DOI:** 10.3390/children5060068

**Published:** 2018-06-01

**Authors:** Alexandra C. Russell, Amanda L. Stone, Andi Wang, Lynn S. Walker

**Affiliations:** 1 Department of Pediatrics, Vanderbilt University School of Medicine, Nashville, TN 37232, USA; lynn.walker@vanderbilt.edu; 2 Department of Pediatrics, Oregon Health and Science University, Portland, OR 97239, USA; stoneam@ohsu.edu; 3 Department of Psychology, Vanderbilt University, Nashville, TN 37235, USA; Andi.wang@vanderbilt.edu

**Keywords:** chronic nausea, functional abdominal pain, nausea severity scale

## Abstract

The objective of this study was to develop a pediatric measure of chronic nausea severity, the Nausea Severity Scale (NSS), and evaluate its reliability and validity in youth with abdominal pain-related functional gastrointestinal disorders (AP-FGID). Pediatric patients (aged 11–17 years-old, *n* = 236) presenting to an outpatient clinic for evaluation of abdominal pain completed the NSS, Children’s Somatization Inventory (CSI), Functional Disability Inventory (FDI), Abdominal Pain Index (API), Patient-Report Outcomes Measurement Information System (PROMIS), Anxiety and Depression Scales and the Pediatric Rome III Questionnaire for FGIDs. The NSS demonstrated good concurrent, discriminant, and construct validity, as well as good internal consistency. One-third (34%) of AP-FGID patients reported experiencing nausea “most” or “every day” in the previous two weeks. The severity of nausea was higher in females than males and correlated significantly with the severity of somatic symptoms, functional disability, anxiety, and depression. The NSS is a valid and reliable measure of nausea in children with AP-FGID.

## 1. Introduction

Nausea is a common symptom that accompanies many acute pediatric illnesses and typically resolves with treatment and resolution of illness [1]. Chronic or recurrent nausea without identifiable etiology, in contrast, may be refractory to treatment [2]. Nausea is particularly common in pediatric patients with abdominal pain-related functional gastrointestinal disorders (AP-FGID) and is estimated to affect between 45% [3,4] and 85% [5] of this patient population. A study of pediatric patients with AP-FGIDs found that half experience nausea at least twice a week and more than a quarter experience nausea daily [4]. In youth with AP-FGIDs, the presence of nausea has been associated with greater disability, fatigue, anxiety, and negative effect and is rarely explained by identifiable organic etiology [2,3,6,7]. Further, a recent study found youth with AP-FGIDs and nausea, compared to youth with AP-FGIDs alone, reported more severe gastrointestinal, extra-intestinal, and internalizing symptoms (anxiety and depression) [3]. These differences between youth with and without nausea in the context of AP-FGIDs remained significant when accounting for abdominal pain severity. Thus, chronic nausea in youth with AP-FGIDs represents a unique risk factor for poor health outcomes [3]. 

Two measures of pediatric nausea have been used to assess acute nausea severity, but neither is appropriate for assessment of chronic nausea as they do not assess the frequency, duration, or chronicity of nausea episodes over time. Specifically, the Nausea Profile has been validated for pediatric assessments, but does not assess frequency or chronicity [7,8], and the Baxter Retching Faces (BARF) scale assesses the severity of pediatric nausea but has only been validated for acute nausea [9]. A validated scale to assess the severity of chronic nausea in children would facilitate research to further understanding and improve treatment of this debilitating condition. 

The purpose of this study was to develop a pediatric patient-report measure of chronic nausea and to evaluate its reliability and validity in a sample of pediatric patients with AP-FGIDs. 

## 2. Materials and Methods

### 2.1. Instrument Development

The Nausea Severity Scale (NSS) was developed by the authors, a multidisciplinary team comprised of pediatric psychologists and gastroenterologists. The Abdominal Pain Index (API), a validated pediatric patient-report measure of the severity of chronic abdominal pain [10], served as a model for the NSS. The NSS assesses four characteristics of nausea during the prior two weeks: number of days with nausea, number of nausea episodes per day, typical nausea duration, and typical intensity of nausea episodes. A total score represents the severity of nausea. The NSS is freely available in the Supplemental Material and can be used without cost if properly cited.

### 2.2. Participants

Participants were adolescents with AP-FGIDs between the ages of 11 and 17 years (*n* = 236). All participants were enrolled in an ongoing IRB-approved, randomized controlled trial evaluating the effect of online cognitive behavioral therapy on health outcomes in AP-FGID patients (R01 HD076983). The study was approved by the Institutional Review Board of Vanderbilt University Medical Center (23 April 2014). Participants were consecutive new patients who presented to the pediatric gastroenterology clinic of a tertiary care center for evaluation of abdominal pain of >2-month duration and no prior diagnosis of explanatory organic disease. Patients were eligible for participation if no significant current organic disease was identified in the medical evaluation and they had internet access. Exclusion criteria included the presence of chronic disease which may affect gastrointestinal symptoms, non-English speaking, inability to read at the 6th grade level, or not having a participating parent/guardian. The analyses reported here are based on baseline (pretreatment) data from study participants enrolled from November 2014 through May 2017. 

### 2.3. Procedure

A research staff member approached patients and their parents in the clinic who had indicated interest in the study. Patients were screened for eligibility and if eligible, informed assent was obtained from adolescents and informed consent was obtained from parents. Patients and parents completed questionnaires on iPads during their clinic visit. Study data were collected using REDCap (research electronic data capture) [11], a secure, web-based application designed to support data capture for research. Medical records were reviewed for results of the medical evaluation to determine exclusion for organic disease.

### 2.4. Measures

#### 2.4.1. Nausea Severity Scale (NSS)

The NSS assesses the frequency, duration, and intensity of nausea experienced in the past two weeks. The format of the NSS is similar to that of the API [10], a measure that assesses the severity of chronic abdominal pain. The first three items ask participants to rate how often they had nausea (0 = *not at all*, 4 = *every day*), how many times a day they had the nausea (0 = *none*, 4 = *constant during the day*), and how long the nausea lasted (0 = *no nausea*, 4 = *most or all of the day*). An additional item asks participants to rate the typical intensity of their nausea using a 0–10 rating scale where 0 = *no nausea* and 10 = *the MOST nausea possible*. The NSS was scored similarly to the API, in that the only item not on a five-point scale (item 4) was converted to a five-point scale ranging from 0 to 4. The mean of all four items yields a total score ranging from 0 to 4.

#### 2.4.2. The Children’s Somatization Inventory (CSI)

The CSI is a self-report measure of the intensity of 24 somatic symptoms (e.g., headaches, low energy, dizziness, chest pain) during the past two weeks [12]. Participants rated how much they were bothered by each symptom using a five-point scale ranging from 0 = *not at all* to 4 = *a whole lot*. Subscale scores were computed for gastrointestinal (GI) symptoms and non-GI symptoms by averaging the relevant items for each subscale. For the purposes of this study, the question regarding nausea was excluded from the GI symptom subscale. Both subscales had good internal consistency; Cronbach α-coefficients were 0.736 and 0.902 for the GI and non-GI symptom subscales, respectively. 

#### 2.4.3. Functioning

The Functional Disability Inventory (FDI) assesses the individual’s perception of difficulty in physical and psychosocial functioning due to physical health during the previous two weeks [13,14]. Participants reported the degree of difficulty of performing 15 activities, scored on a five-point scale ranging from 0 = *no trouble* to 4 = *impossible*. Items were averaged to compute a composite score. In this sample, the Cronbach α-coefficient for the FDI was 0.875.

#### 2.4.4. Abdominal Pain

The API assesses the frequency, duration, and intensity of abdominal pain experienced during the past two weeks [10]. The frequency of abdominal pain episodes during the two-week period was rated on a six-point scale reported on a scale from 0 = *not at all* to 5 = *every day*. The frequency of abdominal pain episodes during a typical day was rated on a six-point scale ranging from 0 = *none* to 5 = *constant during the day*. Participants rated the typical duration of pain episodes on a nine-point scale ranging from 0 = *none* to 8 = *all day* and the typical intensity of abdominal pain on an 11-point scale from 0 = *none* (0) to 10 = *the most pain possible*. The four items of the API are used to calculate a composite score using the techniques described previously by this group [10]. This is converted to a five-point scale to yield a mean ranging from 0 to 4. The Cronbach α-coefficient for the API was 0.777.

#### 2.4.5. The Patient-Reported Outcomes Measurement Information System (PROMIS) Anxiety and Depression Scales

The Pediatric PROMIS Anxiety and Depression Short-Forms assess the severity of anxiety and depressive symptoms in the past week [15]. Participants rated the frequency of eight anxiety and eight depressive symptoms on a five-point scale ranging from 0 = *never* to 4 = *almost always*. Items were summed, and total scores ranged from 0 to 32 for each scale. The Cronbach α-coefficient values were 0.939 and 0.966 for the PROMIS anxiety and depressive symptom scales, respectively.

#### 2.4.6. Functional Gastrointestinal Disorders

The Pediatric Rome III Diagnostic Questionnaire for FGIDs assesses symptoms associated with the ROME III diagnostic criteria. Participants completed 24 items to assess symptom criteria for irritable bowel syndrome (IBS) and functional dyspepsia (FD) [16]. Participants’ responses were coded for the presence versus absence of IBS and FD.

## 3. Results

### 3.1. Demographic Characteristics

The sample included 236 adolescents with AP-FGIDs between the ages of 11 and 17 years (Table 1). The average age of participants was 13.93 years-old (standard deviation (SD) = 1.87 years) and the majority were female (62.3%, *n* = 147). The majority of participants were Caucasian (84.3%, *n* = 199). Participants also included those who were African American (8.1%, *n* = 19), American Indian (1.3%, *n* = 3), Asian (0.4%, *n* = 1), or other/mixed race (5.1%, *n* = 13). Only 5.1% (*n* = 12) identified as Hispanic. 

### 3.2. Descriptive Statistics for Items on the Nausea Severity Scale

Frequency statistics for each of the four items on the NSS are presented in Table 2. One-third (34%) of AP-FGID patients reported nausea on “most” or “every day” in the past two weeks. Nausea lasted “most or all of the day” for 11% of AP-FGID patients. Among those endorsing nausea at least one or two days over the past two weeks (*n* = 189), the average intensity rating was 4.69 (SD = 2.24, median = 5.00, range = 0–10) and mean for the NSS was 2.01 (SD = 0.88, median = 1.90, range = 0.25–4.00). Percentiles for mean scores on the NSS for pediatric AP-FGID patients endorsing nausea at least one or two days over the past week were as follows: 10th percentile = 0.95, 25th percentile = 1.30, 50th percentile = 1.90, 75th percentile = 2.60, 90th percentile = 3.35. For the total sample, including both patients with and without nausea, the mean for the NSS was 1.62 (SD = 1.11, median = 1.60, range = 0.00–4.00). 

Females reported significantly greater severity of nausea on the NSS (mean = 1.82, SD = 1.13) compared to males (mean = 1.31, SD = 1.03), *t* (234) = 3.45, *p* < 0.001. Nausea severity had a low but significant correlation with age, *r* = 0.15, *p* < 0.02.

### 3.3. Internal Consistency

The NSS had high internal consistency, as indicated by Cronbach’s α-reliability coefficient for the total NSS score which had a value of 0.879.

### 3.4. Concurrent Validity

Concurrent validity of the NSS was evaluated by examining the correlation of the NSS with the Nausea item of the CSI. Following convention [17], we defined a large effect as a Pearson’s correlation coefficient of between 0.4 and 0.6, and a moderate effect as a Pearson’s correlation between 0.2 and 0.4. The NSS score strongly correlated with ratings for the item on the CSI that assesses nausea (*r* = 0.670, *p* < 0.001). 

### 3.5. Construct Validity

Construct validity was evaluated by examining the correlation of the NSS with related patient-report measures. Our previous work showed that AP-FGID patients who reported nausea at their initial pediatric gastroenterology evaluation reported significantly greater extra-intestinal somatic symptoms, depression, and anxiety than those who presented without nausea [3]. Therefore, we anticipated that the NSS would correlate significantly with measures of GI and non-GI somatic symptoms (CSI-GI, CSI-non-GI), functional disability (FDI), abdominal pain severity (API), depression (PROMIS depression), and anxiety (PROMIS anxiety). Observed Pearson correlations between these measures and NSS total scores at baseline are reported in Table 3. 

The NSS was strongly correlated with CSI-GI symptoms (*r* = 0.55, *p* < 0.001), CSI-non-GI symptoms (*r* = 0.45, *p* < 0.001), and with FDI disability scores (*r* = 0.42, *p* < 0.001). The NSS also correlated significantly with PROMIS depression (*r* = 0.26, *p* < 0.001) and PROMIS anxiety (*r* = 0.26, *p* < 0.001). Correlations remained significant after controlling for level of abdominal pain (see Table 4).

## 4. Discussion

This study evaluated the psychometric properties of a patient-report measure of chronic nausea severity, the NSS, in a clinical sample of pediatric patients with AP-FGIDs. The NSS demonstrated good internal consistency, that is, the items cohered with each other in assessing the unique construct of nausea severity. The validity of the NSS was evident in its high correlations with the single item measure of nausea severity on the CSI and with measures of other symptoms known to be associated with nausea, including somatic symptoms, functional disability, anxiety, and depression. Thus, the NSS appears to be reliable and appropriate for research on nausea in children with AP-FGIDs.

Study results also contribute to our understanding of nausea in pediatric AP-FGIDs. Specifically, the finding that the correlation between the NSS and measures of somatic and psychological symptoms remained significant when controlling for level of abdominal pain indicates that nausea is a unique correlate of somatic and psychological symptoms in AP-FGID patients, above and beyond the severity of their abdominal pain. These findings are consistent with those from our previous work with a large, prospective cohort of 871 adolescent patients with AP-FGID in which we found that AP-FGID patients with significant nausea, compared to those with AP-FGIDs alone, had more depressive symptoms at initial medial evaluation and were significantly more likely to meet Diagnostic and Statistical Manual of Mental Disorders-Fourth Edition (DSM-IV) criteria for an anxiety or depressive disorder at follow-up [3]. Findings of the present study also are consistent with work by Kovacic and Levy showing that nausea severity correlated significantly with disability, anxiety, and maladaptive coping in children with AP-FGIDs [4,18].

Nausea Severity Scale scores had a low but significant correlation with age and were significantly higher for females than males. This is in line with previous studies that have shown that pediatric patients with AP-FGIDs who report nausea are slightly older than those who do not report nausea [3] and that chronic nausea is more common in females [9]. 

A limitation of this study is the lack of a comparison group of healthy youth without AP-FGIDs. The NSS will need to be tested for discriminant validity in future studies using a healthy control group. It will also need to be tested among children with nausea as their predominant symptom, in accordance with the new Rome IV criteria for functional nausea [19]. Another limitation of the present study is the lack of consistent medical evaluation for secondary conditions such as postural orthostatic tachycardia syndrome (POTS) which may be associated with chronic nausea. Although all youth with AP-FGIDs received an extensive medical evaluation through a pediatric gastroenterology clinic, further medical evaluations by other subspecialties were at the discretion of the physician and not systematically tracked as a part of the current study. Given recent evidence that youth with functional disorders with POTS and youth with functional disorders without POTS show similar rates of comorbid conditions such as nausea [20], it is unlikely the current findings are explained by a secondary condition.

The NSS demonstrated adequate psychometric properties for the measurement of nausea in adolescents with AP-FGIDs. This is the first study to assess the psychometric properties of a chronic nausea measure in this population. Further validation of this instrument is warranted to enable its use on a larger scale and to validate the new Rome IV diagnostic criteria for functional nausea. Nausea is the most frequent gastrointestinal complaint among children with IBS and correlates with markers of physical and psychological health above and beyond abdominal pain. A thoroughly validated assessment scale will assist advancement in clinical research and provide a reliable measure for therapeutic trials. 

## Figures and Tables

**Table 1:** Demographic characteristics.

Demographics	Pediatric AP-FGID Patients (*n* = 236)
Sex, *n* (%)	
Female	147 (62.3%)
Male	89 (37.7%)
Age, *Mean ± SD, years*	13.93 ± 1.87
Race, *n* (%)	
Caucasian	199 (84.3%)
African American	19 (8.1%)
American Indian	3 (1.3%)
Asian	1 (0.4%)
Hispanic	12 (5.1%)
Other/mixed race	13 (5.1%)

AP-FGID**,** abdominal pain-related functional gastrointestinal disorders; SD, standard deviation.

**Table 2:** Item level frequencies for the Nausea Severity Scale (NSS).

Item Response	*n* (%)
*Nausea Frequency (Item 1)*	
Not at all	45 (19.1)
One or two days	70 (29.7)
Three or four days	40 (16.9)
Most days	47 (19.9)
Every day	34 (14.4)
*Daily Frequency of Nausea Episodes (Item 2)*	
None	57 (24.2)
Once a day	65 (27.5)
Two or three times a day	72 (30.5)
Four or more times a day	19 (8.1)
Constant during the day	22 (9.3)
*Nausea Duration (Item 3)*	
No nausea	48 (20.3)
Less than 30 min	61 (25.8)
Half an hour to an hour	69 (29.2)
One to 4 h	30 (12.7)
Most or all of the day	27 (11.4)
*Typical Nausea Intensity (Item 4)*	
No nausea (0)	50 (21.2)
1	6 (2.5)
2	24 (10.2)
3	26 (11.0)
4	29 (12.3)
5	30 (12.7)
6	26 (11.0)
7	23 (9.7)
8	12 (5.1)
9	4 (1.7)
The most nausea possible (10)	4 (1.7)

**Table 3:** Pearson correlations between self-reported variables and NSS.

	NSS	CSI-GI	CSI-Non-GI	FDI	API	PROMIS Depression	PROMIS Anxiety
CSI-GI symptoms	0.55 *	-					
CSI-Non-GI symptoms	0.45 *	0.63 *	-				
FDI	0.42 *	0.54 *	0.55 *	-			
API	0.42 *	0.50 *	0.39 *	0.51 *	-		
PROMIS depression	0.26 *	0.35 *	0.46 *	0.33 *	0.18 *	-	
PROMIS anxiety	0.26 *	0.37 *	0.44 *	0.34 *	0.23 *	0.77 *	-
Mean	1.62	1.25	0.92	1.07	2.26	7.51	11.22
SD	1.11	0.65	0.67	0.74	0.88	8.39	8.49

* *p* < 0.01; NSS, Nausea Severity Scale; CSI Children’s Somatization Inventory; FDI Functional Disability Inventory; API Abdominal Pain Index; GI, gastrointestinal; PROMIS, Patient-Reported Outcomes Measurement Information System; SD, standard deviation.

**Table 4:** Pearson correlations between self-reported variables and NSS, controlling for abdominal pain.

	NSS	CSI-GI	CSI-non-GI	FDI	PROMIS Anxiety	PROMIS Depression
CSI-GI symptoms	0.43 **	-				
CSI-Non-GI symptoms	0.34 **	0.55 **	-			
FDI	0.27 **	0.37 **	0.44 **	-		
PROMIS Anxiety	0.19 *	0.30 **	0.39 **	0.26 **	-	
PROMIS Depression	0.21 **	0.30 **	0.44 **	0.28 **	0.77 **	-

* *p* < 0.01, ** *p* < 0.001; NSS, Nausea Severity Scale; CSI, Children’s Somatization Inventory; FDI, Functional Disability Inventory; API, Abdominal Pain Index, GI, gastrointestinal; PROMIS, Patient-Reported Outcomes Measurement Information System.

## Supplementary Materials

The following are available online at http://www.mdpi.com/2227-9067/5/6/68/s1, The Nausea Severity Scale (NSS).
