# Supplementary material for: Development and Validation of a Nausea Severity Scale for Assessment of Nausea in Children with Abdominal Pain-Related Functional Gastrointestinal Disorders
Source: Children (Basel). 2018 Jun 1;5(6):68. doi: 10.3390/children5060068 (PMC6025273; doi:10.3390/children5060068)
Supplement: Supplementary file 1 [file children-05-00068-s001.pdf]

The Nausea Severity Scale (NSS)

1. In the last 2 weeks, how often have you had nausea (feeling like you might throw-up)?

    0. Not at all
    1. One or two days
    2. Three or four days
    3. Most days
    4. Every day

2. In the last 2 weeks, how many times a day did you usually have the nausea?

    0. None
    1. Once a day
    2. Two or three times a day
    3. Four or more times a day
    4. Constant during the day

3. In the last 2 weeks, when you had nausea, how long did it last?

    0. No nausea
    1. Less than 30 minutes
    2. Half an hour to an hour
    3. One to four hours
    4. Most or all of the day

4. In the last 2 weeks, when you had the nausea, how much did you usually have?

    No Nausea 1 2 3 4 5 6 7 8 9 The most nausea possible
